# Could anakinra outmatch dexamethasone/tocilizumab in COVID-19?

**DOI:** 10.1186/s42269-022-00781-5

**Published:** 2022-04-13

**Authors:** Rahul Gupta

**Affiliations:** Department of Microbiology and Immunology, Stony Brook University, Stony Brook, NY USA

**Keywords:** Anakinra, COVID-19, IL-1, suPAR, Dexamethasone, Tocilizumab, NLRP3, Cell death, Neutrophil migration

## Abstract

The hyperinflammatory state leading to an aberrant cytokine production, culminating in acute respiratory distress syndrome, sepsis and multi-organ dysfunction contribute much to the pathophysiologies of severe COVID-19. These severe patients have similar clinical manifestations with patients suffering from certain auto-inflammatory disorders and cytokine storm syndromes. Interestingly, anakinra (blocking both IL-1α and IL-1β) has shown promises in treating these patients with hyperinflammatory disorders, sepsis with multiorgan failures. Another inflammasome, AIM2, involved in production of IL-1 has also been found to be implicated in COVID-19. IL-1β, a known procoagulant, causes induction of tissue factor with increasing vascular endothelial permeability loss ensuing in hypercoagulability-one of the cardinal features of the disease. Hence, anakinra a 17kD recombinant human IL-1 receptor antagonist, used widely in Rheumatoid Arthritis treatments might prove efficacious in attenuating the hyperinflammatory state of the disease. Indeed, some of the controlled clinical trials have shown anakinra to effectively decrease mortality and hospital stay. Targeted cytokine blocking are always preferable in comparison with non-specific blocking (steroids) as it is more restrained with the chances of dampening of systemic immune system being much less. Early cell death and neutrophil migration have been one of the pivotal events in COVID-19 pathogenesis. Hence, suPAR levels which measures IL-1α (necroptosis) and S100A8/A9 (neutrophil migration) can perhaps be a good early biomarker predicting the disease progression. Lastly and importantly, as the vaccines are raised against spike protein and the different variants of concern are known to evade the neutralizing antibodies by varying degrees, it will be deserving to assess anakinra, against the variants of concern as an immunomodulatory drug.

**To the Editor:**

## Background

The attenuation of the hyperinflammated state has been thought as a measure to contain the pathophysiologies of severe COVID-19.These severe patients share similar phenotypes with patients suffering from autoinflammatory disorders like- adult onset still’s disease and familial mediterranean fever (Cavalli et al. 2020; Huet et al. 2020). Also, patients suffering from cytokine storm syndromes with hyperinflammations like- macrophage activation syndrome, haemophagocytic lymphohistiocytosis and chimeric antigen receptor T-cell (CART) mediated cytokine release syndrome share clinical manifestations with severe COVID-19 patients (Cavalli et al. 2020; Huet et al. 2020). As a result, various clinical trials with non-selective cytokine inhibition by glucocorticoids (dexamethasone) and selective targeted cytokine inhibition (blocking IL-1, IL-6, GM-CSF, etc.) are being pursued for managing COVID-19(https://www.covid-trials.org). Initially, the concept of involvement of IL-1 in the pathophysiology of COVID-19 was little sceptical and underappreciated, as IL-1 was not found to be highly activated in patients. IL-1 is a notoriously known cytokine, for its propensity toward degradation, makes it difficult to analyze from patient’s sample. But now, compelling evidences from different laboratories have shown IL-1 activation and NLRP3/AIM2 inflammasome involvement in the pathogenesis of the disease (Junqueira et al. 2021; Vora et al. 2021) with anakinra having a benefiting effect (Cavalli et al. 2020; Huet et al. 2020; Kyriazopoulou et al. 2021a, b; Vora et al. 2021).

## Main text

Both dexamethasone and tocilizumab (anti-IL-6R), are powerful immunosuppressants known for excessive dampening of immune system leading to downscaling of Procalcitonin (PCT) and CRP levels – ensuing in probable downside of faulty diagnostic capacity detection of secondary bacterial infections (Kooistra et al. 2021). Hence, antibiotic usage and stewardship becomes skewed.

Targeted inhibitions are always preferred in comparison with non-selective inhibitions as it leads to lesser degree of immunosuppression with ensuing secondary infections (Hooftman and O’Neill 2021). Dexamethasone, being a NF-κB inhibitor, by IκBα activation (Auphan et al. 1995), broadly inhibits a wide array of NF-κB dependent pro-inflammatory cytokines like- IL-1, IL-2, IL-6, 1L-8, IL-18, TNF-α, CXCL1, CXCL10, Rantes, etc. (Adcock 2001; Hooftman and O’Neill 2021). The rise of mucormycosis events in India further substantiates the immunosuppression effects of corticosteroids, especially in diabetic patients (Hooftman and O’Neill 2021). More over, glucocorticoids being known to induce hyperglycemia by provoking insulin resistance and beta cell dysfunctioning could lead to exacerbating the comorbid diabetic condition (Suh and Park 2017).

Even, inhibitory effect of dexamethasone can partially be attributed to NLRP3 inflammasome inhibition (Hooftman and O’Neill 2021). More so, with IL-1 working upstream of IL-6 (Kyriazopoulou et al. 2021a), it perhaps makes a worthy approach to target IL-1 blocking for the diseased state by usage of anakinra (which is dual blocker of IL-1α and IL-1β). The recent SAVE-MORE trial guided by soluble urokinase plasminogen activator receptor ( suPAR levels ≥ 6 ng/ml), showed anakinra to have mortality benefits and less secondary infections- by inhibiting IL-6, CRP and lymphocyte counts, even with dexamethasone co-administration (Kyriazopoulou et al. 2021b). suPAR, a biomarker indicative of presence of alarmins like- neutrophil migration promoting calprotectin (S100A8/A9) and IL-1α, is activated earlier than CRP and IL-6 (Kyriazopoulou et al. 2021b). Interestingly, in the CAN-COVID trial, canakinumab (specific monoclonal antibody against IL-1β) failed to show survival benefits (Kyriazopoulou et al. 2021b). This can perhaps be explained by the necessity of IL-1α in the pathogenesis phenomena. IL-1α is known as one of the crucial cytokines liberated initially from lung necrotic cells, which further helps in amplifying the inflammatory loop (Gupta 2020a). With amplification, extended IL-1 family members like IL-36γ and IL-33 cytokines can be produced from the necrotic cells (Martin 2016). IL-36γ is known to inhibit immunoregulatory Treg cell development (Harusato et al. 2017), which could lead to exacerbation of the diseased state. Furthermore, IL-33 induces IL-10 production (Sattler et al. 2014) and enhanced IL-10 has been correlated with increasing IFN-γ producing CD4^+^/CD8^+^T cells and PD1^+^/Tim3^+^ population – ensuing in T cell exhaustion in severe COVID-19 patients (Lu et al. 2021). Hence, IL-1 can play a central role in upheavaling the immune system in severe patients.

With COVID-19 being characterized by hypercoagulability, IL-1α can play a contributory role to coagulation phenomena too- by itself getting thrombin activated and inducing platelet productions (Burzynski et al. 2019). Simultaneously, with complement factors known to be activated in COVID-19 (Ma et al. 2021) and also a priming agent for NLRP3 signaling (Niyonzima et al. 2020), the diseased state can be thought as an interplay between IL-1 and complement system. Interestingly, aberrent cytokine release with concurrent neurological defects are known bystander effects of CART therapy with IL-1/IL-6 production from monocytes contributing to it (Norelli et al. 2018). Intriguingly, tocilizumab failed to protect mice from neurological damages (meningeal inflammation), while anakinra protected mice both from cytokine release and neurotoxicity (Norelli et al. 2018). With COVID-19 patients known to manifest neurological symptoms and NLRP3 known to be implicated in neurological diseases like Alzheimer’s (Heneka et al. 2013)—blocking IL-1 can conceivably be more efficacious than IL-6 in managing COVID-19. Lastly, the cytokine release induced neurological defects can also shed some light in understanding the long COVID-19 pathology.

## Conclusions

Hence, anakinra will perhaps have an edge over IL-6 and dexamethasone with more restrained and specific immunosuppressing capacity by taming the hyperinflammated and hypercoagulable states. The staggering 10 days half-life of tocilizumab in comparison with 4–6 h for anakinra, further substantiates it. More so, with neutrophil migration and cell death (IL-1α) contributing much to COVID-19 pathology (Gupta 2020a, b), suPAR can be a better early biomarker than non-specific markers (PCT/CRP) for COVID-19 progression.

## Data Availability

Literature survey.

## Abbreviations

IL-1 α/β/2/6/8/18/33/36: Interleukin 1 alpha/beta /2/6/8/33/36.
CART: Chimeric Antigen Receptor T-cell
suPAR: Soluble urokinase plasminogen activator receptor.
NLRP3: Nod Like Receptor family pyrin domain containing 3
AIM2: Absent in melanoma 2
anti-IL-6R: Anti-Interleukin 6 receptor
PCT: Procalcitonin
CRP: C-reactive protein
NF-κb: Nuclear factor kappa-light-chain-enhancer of activated B cells
IκBα: Nuclear factor of kappa light polypeptide gene enhancer in B-cells inhibitor, alpha
TNFα: Tumor necrosis factor-α
CXCL1/10: C-X-C motif chemokine ligand 1/10
SAVE-MORE: suPAR-guided Anakinra treatment for Validation of the risk and Early Management Of severe respiratory failure by COVID-19
CAN-COVID: Efficacy testing of canakinumab in patients hospitalized with severe COVID-1
IFN-γ: Interferon gamma
PD-1: Programmed Cell Death Protein 1
Tim3: T cell immunoglobulin and mucin domain-containing protein 3
